# Perspectives regarding cannabis use: Results from a qualitative study of individuals engaged in substance use treatment in Georgia and Connecticut

**DOI:** 10.1016/j.dadr.2024.100228

**Published:** 2024-03-24

**Authors:** Charles A. Warnock, Ashlin R. Ondrusek, E. Jennifer Edelman, Trace Kershaw, Jessica L. Muilenburg

**Affiliations:** a Center for Interdisciplinary Research on AIDS, Yale School of Public Health, New Haven, CT, USA; b Department of Social and Behavioral Sciences, Yale School of Public Health, New Haven, CT, USA; c Department of Psychology, University of Nebraska-Lincoln, Lincoln, NE, USA; d Yale Program in Addiction Medicine, Yale School of Medicine, New Haven, CT, USA; e Department of Internal Medicine, Yale School of Medicine, New Haven, USA; f Department of Health Promotion and Behavior, University of Georgia College of Public Health, Athens, GA, USA

**Keywords:** Cannabis, Substance use treatment, Qualitative, Beliefs, Attitudes

## Abstract

**Objective:**

Cannabis use is increasingly pervasive throughout the U.S. People in treatment for substance use disorders (SUD) may be especially at-risk of harm due to this changing context of cannabis in the U.S. This study’s objective was to qualitatively describe experiences and beliefs around cannabis among people who had entered treatment for any SUD in the past 12-months.

**Methods:**

From May to November of 2022, we conducted 27 semi-structured interviews (n=16 in Georgia, n=11 in Connecticut) with individuals in treatment for SUD in Georgia and Connecticut. Interviews were recorded, transcribed, and thematically analyzed using an emergent approach.

**Results:**

All participants had used cannabis in the past. Four themes emerged from the interviews. Participants: (1) perceived cannabis as an important contributor to non-cannabis substance use initiation in adolescence; (2) viewed cannabis as a substance with the potential to improve health with fewer side effects than prescription medications; (3) expressed conflicting opinions regarding cannabis as a trigger or tool to manage cravings for other non-cannabis substances currently; and 4) described concerns related to negative legal, social service, and treatment-related consequences as well as negative peer perception relating to the use of cannabis.

**Conclusion:**

Although participants described cannabis’s important role as an initiatory drug in adolescence and young adulthood, many felt that cannabis was a medicinal substance for a range of health challenges. These findings suggest SUD treatment clinicians should address medicinal beliefs related to cannabis among their clients and emphasizes the need for research on cannabis use and SUD treatment outcomes.

## Highlights

• People in SUD treatment associate cannabis with the initiation of substance use in adolescence and early adulthood.
• Some people in SUD treatment see cannabis as a trigger for other substance use.
• Others in SUD treatment believe cannabis may be a beneficial, medicinal substance.
• People in SUD treatment noted using cannabis risked treatment-related consequences like involuntary treatment separation.

## 1 Introduction

Existing research suggests that cannabis use is likely to persist during the early phases of treatment for substance use disorder (SUD) (Hermann et al., 2005, Lake and St. Pierre, 2020, Scavone et al., 2013). This pattern of persistent use is a concern as it may be associated with low treatment efficacy and poor non-cannabis substance use outcomes. Among individuals with opioid use disorder (OUD), persistent cannabis use has previously been linked to an increased risk of premature treatment discontinuation, discontinuation of MOUD persistent injection drug use, and return to opioid use (Franklyn et al., 2017, Levine et al., 2015, Wasserman et al., 1998, Zielinski et al., 2017, Budney et al., 1998). Similarly, individuals with an alcohol use disorder (AUD) who continue to use cannabis during treatment are more likely to have higher levels of alcohol use and fewer alcohol abstinent days in comparison to individuals with AUD who abstain from cannabis during treatment (Weinberger et al., 2016). Treatment programs have varied in their approaches to cannabis among their clients. Traditionally, the use of illegal or non-medical substances while enrolled in addiction treatment programs can result in consequences like administrative discharge from the program (Williams, 2016). However, the legal and societal landscape around cannabis has changed to be more permissive with the legalization of recreational cannabis and pervasive permissive attitudes towards cannabis in the U.S. Treatment centers are in a challenging position when it comes to determining their approach to clients who use cannabis.

As popular opinion evolves to regard cannabis as a less harmful or even medicinal substance, perceptions among clinicians and individuals in treatment for SUD may similarly be shifting towards the view that cannabis use is inconsequential or perhaps even beneficial. This perspective of cannabis stems largely from a harm reduction-rooted philosophy to treatment, in which it is argued that individuals who regularly use substances considered more harmful than cannabis may opt for cannabis instead (Humphreys and Saitz, 2019, Mikuriya, 2004, Valleriani et al., 2020, Adinoff and Cooper, 2019). Indeed, some reasons commonly cited by people who use cannabis and are trying to reduce or stop using drugs other than cannabis include to subjectively ease withdrawal symptoms or as a substitute in place of substances with greater perceived harm potential (Lau et al., 2015, Bergeria et al., 2020). However, considering some research showing worse SUD treatment outcomes among individuals who co-use cannabis with other substances in comparison to individuals who do not co-use cannabis with other substances, the use of cannabis may involve greater risk among this population than among non-treatment seeking individuals. To date, little research has qualitatively examined cannabis use among individuals in treatment for SUD in this new societal context, and the extant qualitative research around cannabis and SUD treatment focuses on addiction professionals who treat adolescents (Sobesky and Gorgens, 2016). This study is among the first qualitative investigations in this new cannabis-permissive cultural context within the United States among adult individuals in active substance use treatment. Understanding the lived experiences and perspectives among individuals in treatment in their own words, rather than purely quantitatively or through the perspectives of clinicians and other treatment providers, could offer fresh research avenues around the context and safety of cannabis use and its effect on treatment and substance use outcomes as well as identify potential strategies for debunking prevalent beliefs within this population by first elucidating them.

As cannabis becomes more prevalent in the general population and perceptions of harm around cannabis decrease, more individuals in treatment for SUD are likely to have positive attitudes and beliefs towards cannabis and its use. The purpose of this qualitative study is to fill this gap by gaining insights regarding the beliefs, perceptions, and experiences of cannabis among individuals in treatment for SUD.

## 2 Materials and methods

### 2.1 Study overview

This research was part of a larger observational parent study (R01AA025954 Kershaw/Muilenburg) examining the impact of health behaviors, social networks, and geographic settings on treatment outcomes among individuals entering formal treatment for SUD in a community setting and who had also used alcohol within the past year. Criteria for participant eligibility in this parent study and this current study were (1) 18 years old or older, (2) spoke English, (3) entered formal treatment within the previous 12 months, and (4) reported drinking alcohol during the previous 12 months. Participants in this parent study were recruited using a mix of purposive and snowball sampling at community SUD treatment centers in Connecticut (cannabis legal state) and Georgia (cannabis illegal state). Participants in this parent study were offered the opportunity to participate in this qualitative research between May and November of 2022 immediately after study entry. Informed consent for this research was obtained as participants entered this parent study where participants were informed about the opportunity to participate in recorded interviews regarding their behaviors and experiences during treatment. This informed consent applied to both the parent study and this qualitative research. Using cannabis while in treatment was not established as criteria for inclusion or exclusion in this research as individuals who may have not used cannabis since entering treatment are likely to have meaningful perspectives and experiences around cannabis. Sampling was determined to be complete once thematic saturation was reached. Thematic saturation was determined to be achieved by agreement amongst the authors (CW, AO, TK, and JM) following weekly meetings across the time of interview data collection during which emergent themes were discussed.

### 2.2 Data collection

Members of the research team conducted semi-structured tele-interviews using Zoom’s audio recording feature. Only research team members and the participant were present for each tele-interview. Interviews lasted approximately 30 minutes with a range of 11–50 minutes. The interview guide for this study was developed and pilot tested by the research team before data collection began. The interview guide sought to collect and understand perceptions and experiences with cannabis and how they impact an individual treatment and recovery journey (See Appendix A for guide). No changes were made to the interview guide during the interview period.

Prior to the interview, participants completed a survey to assess demographic information, substance use behaviors, and past incarceration history. Each participant received $40 debit cards or Amazon gift cards for participating in the interview. All research activities were approved by the Institutional Review Boards at Yale University and the University of Georgia.

### 2.3 Data analysis

Interviews were transcribed using Trint, an AI-assisted audio transcription service (Trint, 2023). Each transcript was then reviewed by a member of the research team for accuracy, de-identified for confidentiality, and then analyzed line-by-line using a constant comparative method guided by an emergent approach (Charmaz, 2002, Glaser, 1965). Two investigators coded the transcripts collaboratively (CW, AO). A preliminary coding structure and qualitative codebook was created based on interview questions and participant responses (Glaser and Strauss, 1967, Miles and Huberman, 1994). The codebook was edited and agreed upon by the two-person team in an iterative fashion until a final comprehensive codebook was created. The final codebook was then used to code each interview line-by-line individually. The transcripts were then coded collaboratively and comparatively until 100% agreement was met. NVivo Version 14 qualitative data analysis software was used to facilitate qualitative code organization and retrieval (Lumivero, 2023). Codes were then compared across interview text data to yield salient themes. Descriptive frequencies and statistics collected prior to the interviews to characterize the population were calculated using R Version 4.2.2.

## 3 Results

### 3.1 Sample characteristics

We conducted n=27 interviews (n=16 in Georgia and n=11 in Connecticut). Among the participants, most identified as White (70.4%) and Female (55.6%). The majority (71.4%) had a lifetime history of incarceration. All were in active inpatient, outpatient, or residential treatment at the time of the interview. Nearly half (48.1%) reported using cannabis in the previous three months.

Participants, on average, selected three substances that they were receiving treatment to address. Most participants (81.5%) were receiving treatment to address alcohol. More than half (59.2%) were receiving treatment to address crack/cocaine. Slightly less than half (44.4%) were receiving treatment to address amphetamines. Less than half (40.7%) were receiving treatment to address opioid use, and 37.0% were receiving treatment to address cannabis use (See Table 1).

**Table 1:** Demographic characteristics of the sample (N = 27).

	Mean (SD) or N (%)
Age	37.4 (9.4)
Race	
White	19 (74.1%)
Black/African American	6 (22.2%)
Other	1 (3.7%)
Ethnicity	
Hispanic/LatinX	1 (3.7%)
Not Hispanic/LatinX	26 (96.3%)
Gender	
Male	12 (44.4%)
Female	15 (55.6%)
Treatment Modality	
Inpatient	5 (18.5%)
Outpatient	13 (48.1%)
Residential	9 (33.3%)
Past three-month cannabis use	13 (48.1%)
Substances in treatment to address	
Alcohol	22 (81.5%)
Amphetamines	12 (44.4%)
Crack/Cocaine	16 (59.2%)
Opioids	11 (40.7%)
Number of substances in treatment to address	3.2 (2.1)
Incarceration History	
Yes	20 (71.4%)
No	7 (28.6%)

### 3.2 Emergent qualitative themes

We identified and categorized experiences with and perceptions of cannabis to identify four emergent themes. Participants (1) perceived cannabis as an important contributor to other substance use initiation in adolescence and young adulthood; (2) viewed cannabis as a medicinal substance with the potential to improve health with fewer side effects than prescription medications; 3) expressed conflicting opinions as to if cannabis was a trigger for other substance use or tool to manage cravings for other non-cannabis substances while in treatment for SUD; and 4) described concerns related to negative legal, social service, and treatment-related consequences as well as negative peer perception relating to the use of cannabis during treatment for SUD.

#### 3.2.1 Theme 1: contribution of cannabis to other substance use initiation in adolescence and young adulthood

All 27 participants reported during the interview that they had used cannabis at some point in their life. When asked to share about their early experiences with cannabis, participants described first using cannabis as an adolescent. This early cannabis use was tied to social settings and interactions with friends and family members. As one participant described, “*I got some marijuana from my sister and we* [my friends and I] *sat down, and we just smoked like, $20 of weed to ourselves. And we were like 14, maybe 13 or 14 and just got super stoned.”*

The participants described these early social experiences with cannabis as commonly intertwined with alcohol. One participant described their initial experience with both cannabis and alcohol as occurring simultaneously: “*It was the first time I smoked and…my buddy gave me a bottle of orange juice. I chugged the whole thing, and then he got mad at me. And he was like, “That was liquor you idiot!”. So, I ended up drinking the first time on same day I first smoked pot.”* Another participant described how it was common to use cannabis and alcohol simultaneously in a social setting as an adolescent and young adult: *“I don't know if you're familiar with the term "getting twisted", but that was a big thing where you would drink alcohol and smoke. If, you know, you were up at school at a party, people would say "Oh, where's the weed? Let's smoke some weed.”*

When considering their early experiences with cannabis, participants frequently discussed cannabis’s role in initiating the use of other drugs. While the use of cannabis alone was mostly considered *“harmless”* or *“inconsequential”* by participants, many described cannabis’s potential role as a *“gateway”* to other narcotic substances as a risk for later substance use initiation. As one participant said, “*Just once you do this drug* [cannabis] *and then you experience this high… you're going to hang out with somebody else and they might have cocaine and you're going to want to experience that high.* Another participant described how using cannabis was the beginning of their substance use journey: “*I first started smoking weed. I loved it. That was my gateway drug… because that weed was so good, I thought, what else could there be? And it just turned out I just found my addictive personality through weed.”*

#### 3.2.2 Theme 2: cannabis as a medicinal substance

Participants described cannabis as a substance with potential medicinal benefit especially. Much of this medical benefit was attributed to cannabis’s beneficially perceived effects on anxiety, sleep, and appetite. One participant discussed her views of cannabis as a beneficial, medicinal substance: “*I feel like marijuana is medicine… It would be amazing if they would use it for something like anxiety or something like that or to deal with like sleep issues. I think it works well as a mood stabilizer.”* Another participant discussed her current use of cannabis to address anxiety and lack of appetite saying, “*Weed for me does not fall under, you know, a drug. I only just do it, you know, randomly, occasionally for anxiety or when I need to eat.”* Issues like sleep were commonly cited as a reason for the use of other substances like opioids. Some participants described using cannabis in place of opioids to sleep: “*It's really hard for me to sleep. So when I smoke, I can actually, like, go to sleep without, like, using fentanyl.”*

Participants commonly discussed cannabis and its medicinal effects favorably in comparison to prescription mental health medications. One participant discussing the perceived benefit of cannabis on mental health said, “*I take Cymbalta… and marijuana, it's probably the same. It could be the same thing. Maybe not as bad. You know, if you just, like, smoked a little bit, not smoked the whole bunch, it would probably be the same effect as Cymbalta.”* Another participant compared the side effects of cannabis to those commonly attributed to mental health medications: “*I definitely feel like it* [cannabis] *would probably have less side effects than all the different medications… you know, some that come with the whole like “May cause suicidal thoughts” or whatever.”*

#### 3.2.3 Theme 3: conflicting opinions around cannabis as a trigger for other substance use or as a tool to manage cravings for other substances during treatment

Participants had conflicting opinions as to if cannabis itself was an individual trigger for other substance use for them. Some participants said cannabis and its use had no relation to any other substance use. As one participant remarked, “*Marijuana is not a trigger for me. Alcohol and cocaine are my triggers.”* Other participants described cannabis as a substance that was not their priority substance or substance of choice. One participant described her relationship with alcohol in comparison to cannabis: “*It's* [cannabis] *not a thing that I need. but alcohol is something I need. You want to come over and hang out and, you know, you can smoke my pot whatever… but you touch my alcohol and there’s a problem.”* However, other participants who detailed cannabis as a trigger for other substance use perceived the use of any mind-altering substance to be potential trigger. One participant described cannabis as a trigger in this way: “*I love marijuana. I know that it's not my drug of choice. But if I get inebriated in any way, shape, or form, my drug of choice will always come into play. So, I know that I have to stay away from marijuana.”* Several participants described the use of cannabis in their social environments to be triggering. One participant described peer cannabis use in their workplace to be especially triggering towards their desire to drink alcohol, *“Being around it all the time and smelling it and stuff is a little bit tough at work. Because it like it makes the job harder and it like trying to watch cats and get them all in the bathtub at the same time. Which stresses me out and makes me want to drink.”*

A group of participants discussed cannabis’s perceived utility as a tool to manage cravings that trigger the use of other substances for which they were receiving treatment. As one participant discussing cannabis and triggers for substance use said, “*Let's say if I am having some kind of craving and then I do smoke marijuana. The desire to go to the lengths to fulfill that craving are pretty much quelched.”* When specifically discussing cannabis’ effect on substance use treatment goals, some participants expressed that cannabis played a beneficial role in achieving these objectives due to its effectiveness in helping to manage cravings for other substances. One participant remarked, “*I think sometimes it* (cannabis) *helps* (my substance use treatment goals) *in the positive because it will keep me from using other substances.”* Another participant remarked “*I think smoking has like kept me from doing other drugs… It’s really hard for me to sleep without using fentanyl or heroin. So like that’s a really big thing for me. I really don’t smoke* (use cannabis) *unless I’m going to sleep. I’m able to just knock out.”* Reinforcing this association between cannabis and other tools to manage cravings during treatment, another participant compared cannabis to MOUD: *“I think marijuana is helpful during treatment. I mean, no more worse than any MAT* (MOUD) *medication like Suboxone or Methadone.”*

#### 3.2.4 Theme 4: concerns about consequences due to cannabis use

When discussing using cannabis during their treatment for SUD, many participants expressed concerns about treatment-related consequences due to cannabis use. Although only one participant reported experiencing *“trouble”* with their treatment provider after using cannabis, other participants described witnessing peers experience consequences like administrative discharge due to cannabis use: “*There are people who have been discharged in the last month for getting high here.”* When discussing these consequences, participants often described the negative impact that these consequences could have on an individual’s treatment journey. As one participant said, “*If they were to use marijuana here, they would get kicked out which could cause them to go and do something way worse than marijuana.”*

The concern for consequences due to cannabis use expressed by participants were not limited to treatment-related consequences. The majority of participants in this research had a previous history of incarceration and expressed concerns about legal issues related to cannabis use and their probationary or parolee legal status: “*If I knew my probation officer would let me get away with smoking marijuana, I would be smoking today. The only reason I don't, and it's got nothing to do with recovery, it has to do with going back to jail or prison*.” In addition to these legal concerns, participants also discussed the potential for consequences stemming from family and child services issues. As one participant said, “*I do have DFCS* (Department of Family and Child Services) *involved in my life and just knowing that my son's actually still in the state system, I didn't feel comfortable with it* [using cannabis]*. So, I just went ahead and put the vape away.”*

Participants who reported using cannabis during treatment for SUD recognized that using cannabis while participating in treatment settings and activities that emphasize total abstinence could lead to treatment-related consequences, negative peer perceptions, or otherwise “*tarnish my* [the participant’s] *testimony of recovery.”* One participant who used cannabis during treatment discussed using secretly to avoid negative peer perceptions of their recovery journey: *”I know it* [using cannabis] *is frowned upon in the recovery community. So, it is probably the only secret that I have in my life today about anything.”* Another participant discussed “*staying quiet*” about their cannabis use during the course of their treatment program: *”I'm in a 12-step program type situation, so all chemically mind-altering substances and stuff aren't allowed…They think that that will just take you back out into full blown addiction with, you know, the stuff that I was using. I don't believe the same thing.”*

## 4 Discussion

This research found that, while participants associated cannabis with other substance use behavior in adolescence and young adulthood, they had conflicting beliefs around cannabis as a proximal trigger for other substance use. Some participants perceived the inebriating effects of cannabis as a dangerous trigger. Other participants disagreed or described cannabis as a beneficial tool to manage cravings for other substances. A significant portion of participants reported using cannabis to manage issues with anxiety, sleep, and appetite. Surprisingly and despite clinical interest in the use of cannabis to treat issues with pain, participants did not mention pain when discussing reasons for medicinal use of cannabis even though people with a SUD are more likely to experience issues with chronic pain than the general population (Fisher et al., 2021, Manhapra and Becker, 2018). Participants also described concerns about social service, legal, and treatment-related consequences due to cannabis use during treatment, and participants who reported using cannabis detailed secrecy and discretion in relation to their cannabis use to avoid negative peer perception as well as other consequences.

Participants identified young adulthood and adolescence as periods when they started using cannabis. Data from SAMHSA’s Treatment Episode Data Set (TEDS) shows that most people admitted into SUD treatment programs initiate substance use between the ages of 12 and 24 (Substance Abuse and Mental Health Services Administration, 2017). It is similarly unsurprising that participants related cannabis and alcohol as being intertwined during this initiatory period. Alcohol is the second most commonly co-used substance with cannabis after tobacco, and between 20% and 70% of adolescents who used cannabis in the past-year report simultaneous cannabis and alcohol use (Patrick et al., 2018, Schlienz and Lee, 2018, Terry-McElrath et al., 2013).

Participants in this research linked their early use of cannabis to the initiation and development of other problematic substance use later in life. There is evidence within the literature to suggest cannabis acts as an initiator and progressive “gateway” substance leading to other substance use later (Kandel and Faust, 1975, Lynskey and Agrawal, 2018, Secades-Villa et al., 2015, Williams, 2020). Unprompted, several participants used the “gateway drug” term when referring to cannabis’s role on other substance use. This potential gateway effect is likely to be at least partly explained by social processes (Lynskey and Agrawal, 2018). Previous population research examining alcohol and drug initiation sequencing and use progression has found that the initiation of one substance likely results in exposure to risky peer affiliations as well as other risky social structures (Hines et al., 2016, Wagner and Anthony, 2002). In agreement with these studies, participants often described adolescent and young adulthood cannabis use as occurring in social settings with like-minded peers. Prior research also indicates this gateway effect is unlikely to be attributable to the effects of using cannabis alone. Studies examining alcohol, tobacco, and cannabis initiation have lent more towards a shared liability model of substance use where the initiation and progression of any of these substances contributes to the initiation and progression of another (Kendler et al., 2012, Morral et al., 2002).

As U.S. states have legalized cannabis for medicinal purposes, the perception of cannabis as a medicinal substance has become increasingly popular (McGinty et al., 2016). According to this research, this perception of cannabis as a medicinal substance has similarly extended to people who are in treatment for SUD. Participants repeatedly referred to cannabis as a beneficial, medicinal substance with fewer side effects than prescription medications. Several participants reported currently using cannabis to address mental health challenges like anxiety. Individuals who use cannabis recreationally and medicinally commonly claim cannabis is a substance that reduces anxiety symptomology (Mercurio et al., 2019, Osborn et al., 2015). Although some research has found that the use of Δ9-tetrahydrocannabinol-potent cannabis products like those found in the recreational market may acutely relieve anxiety, other research has found that cannabis actually worsens or increases the risk of developing chronic depressive and anxiety disorders (Lev-Ran et al., 2014, Martin et al., 2021, Bahorik et al., 2017). Our findings suggest a need to address these medicinal beliefs around cannabis and mental health among a population who may be more likely to experience harm due to its use.

Contrary to other research tying cannabis to the persistence of other substance use among people in treatment for SUD, our participants were conflicted about cannabis’s relationship to other substance use currently as adults (Mojarrad et al., 2014, Aharonovich et al., 2005). Participants who identified cannabis as a risk for other substance use primarily attributed this risk to cannabis’s intoxicating effects. Notably in opposition to this view, other participants discussed cannabis as a substance that reduces the risk of using opioids by reducing cravings. Initially heralded as an “exit drug” in the wake of falling overdose rates in cannabis legal states immediately post-legalization for recreational purposes, recent research finds those overdose trends to be reversed with legal states experiencing ~20% greater opioid overdose mortality rate than expected in comparison to other states in the U.S (Shover et al., 2019, Bachhuber et al., 2014). Other research in the general population finds that people who use cannabis are less likely to decrease their opioid use over a three-year period in comparison to people who do not use cannabis (Olfson et al., 2018).

However, this perception of cannabis by participants as a beneficial substance with the potential to be used by individuals in treatment for SUD to manage cravings for other non-cannabis substances is intriguing. Cravings management is often a large focus of certain SUD treatments and using cannabis as a cravings management tool or medication remains largely unexplored (National Institute on Drug Abuse, 2018). If cannabis is in fact a beneficial medicine to control cravings as perceived by a substantial group of participants in this research, it may serve to better SUD treatment outcomes among individuals engaged in SUD treatment who use cannabis. However, other participants found personal use of cannabis or cannabis use in their social environment to trigger cravings for other substances. While personal use of cannabis may be beneficial to some individuals in treatment, this research suggests that it has the potential to be deleterious to substance use treatment modalities like residential or other sober treatment settings.

Participants expressed concerns related to using cannabis while involved in their substance use treatment programs. Many of these concerns were due to the potential to experience treatment program-related consequences like administrative discharge. Treatment programs usually operate with a plethora of rules and expectations to create a supportive and safe environment for patients, but discharge from treatment services for violations of these rules or expectations can lead to consequences like worsening substance use and total treatment disengagement (Williams, 2016). Both Connecticut and Georgia participants in this research expressed concern for treatment-related consequences due to cannabis use. This may be due to abstinence-only treatment approaches pervasive throughout the U.S. that include cannabis as a substance of addictive concern. This uniform popularity in abstinence-only treatment approaches may have led to invariability in themes across participants in both Georgia and Connecticut. Other participant concerns about cannabis existed around legal and family services. Participants in this research resided in Georgia, a U.S. state that has not legalized cannabis for any purpose, and Connecticut, a U.S. state that only recently legalized cannabis for recreational consumption in 2021.

These findings must be interpreted with several limitations. Participants were engaged in various treatment programs, and we are unable to speak to the policies regarding cannabis and cannabis use at each specific treatment site. The sample was a self-selected convenience sample recruited from active participants in the parent study, and individuals who may have not agreed to participate in both this research study and the parent research study may have different views about cannabis from individuals who did agree to participate in this research. A large proportion of participants were recruited using a snowball sampling method which allowed participants to refer peers to participate in this research. Individuals in treatment for SUD often develop support networks of like-minded peers (Shalaby and Agyapong, 2020). This could have led to the sample consisting largely of similar viewpoints. Most participants were formerly incarcerated. Individuals in treatment for SUD who have not previously been incarcerated may have differing views and less hesitancy regarding using cannabis in the context of potential legal ramifications due to use. Also, our participants were mostly White and non-Hispanic. Individuals in treatment from racially and ethnically minoritized backgrounds may have differing views regarding cannabis use.

This study interviewed individuals in treatment for substance use around their beliefs about, experiences with, and perceptions of cannabis use. Participants related early cannabis use with the development of substance use disorder later in life yet primarily viewed cannabis as a substance with medicinal value to treat mental health challenges. In addition, participants were conflicted towards cannabis as either a trigger for non-cannabis substance use or as a substance with the utility to manage cravings for non-cannabis substances. Participants who did use cannabis during treatment often feared legal or treatment-related consequences due to use. Prior research has well-established the connection between early cannabis (or early substance use in general) and an increased risk of developing substance use challenges later in life as well as the increasingly positive, medicinal beliefs around cannabis within the U.S. at-large (Kendler et al., 2012, McGinty et al., 2016, Morral et al., 2002). Similarly, it is well-known that cannabis use while in treatment can have adverse individual legal and treatment-related consequences (Williams, 2016). Yet, hearing directly from individuals in treatment for SUD has the potential to create new insights. Although previous research has examined the use of cannabis to manage symptoms of withdrawal from other non-cannabis substances, this qualitative investigation suggests a better understanding is needed of the potential utility or non-utility of cannabis as a substance to manage cravings for other non-cannabis substance use (Lau et al., 2015, Bergeria et al., 2020). Efforts should be made to identify individuals in treatment who use cannabis in risky contexts for return to use, and further, if using cannabis alone contributes to poor treatment outcomes or if it is a potential cravings management tool for some individuals in treatment for SUD.

## Funding

This work is funded by 10.13039/100000027NIAAA R01AA025954 and 10.13039/100000025NIMH T32MH020031. No other funding supported this research.

## CRediT authorship contribution statement

**E. Jennifer Edelman:** Writing – review & editing, Methodology, Investigation, Funding acquisition. **Trace Kershaw:** Writing – review & editing, Project administration, Investigation, Funding acquisition. **Charles A. Warnock:** Writing – review & editing, Writing – original draft, Software, Project administration, Methodology, Investigation, Formal analysis, Data curation, Conceptualization. **Ashlin R. Ondrusek:** Writing – review & editing, Project administration, Methodology, Formal analysis. **Jessica L. Muilenburg:** Writing – review & editing, Supervision, Project administration, Methodology, Investigation, Funding acquisition, Conceptualization.

## Declaration of Competing Interest

No conflict of interest was declared.

## Appendix A

During this part of the interview, we are interested in learning how people entering treatment for alcohol or other substances use and perceive marijuana or cannabis in relation to their treatment goals. We will ask you about your marijuana or cannabis use and the use of people you know and how that may affect treatment.

1. How would you describe your treatment goals?
  a. Have they changed since you first entered treatment?
  b. If yes, how have they changed?
2. What do you think about marijuana or cannabis?
  a. Do you have a positive or negative belief or outlook about it?
  b. Why do you think you have that belief or outlook?
3. How does the marijuana or cannabis use of other people in your life or around you affect your use of alcohol or other substances?
4. What did you think about marijuana or cannabis before you entered treatment?
5. Do you know anyone that has used marijuana or cannabis during their treatment? **IF NO SKIP TO BOLD #7**
6. How do you think marijuana or cannabis affects their treatment goals?
7. **Have you ever used marijuana or cannabis? IF NOT SKIP TO BOLD ITALIC**
8. Can you tell me about the first time you used marijuana or cannabis?
  a. How old were you?
  b. Were you alone or with other people?
  c. What did you think about marijuana or cannabis after you tried it the first time?
9. Have you used marijuana or cannabis since you entered treatment? **IF NO, SKIP TO BOLD ITALIC**
10. How do you feel about using marijuana or cannabis?
  a. Do you think it’s a positive or negative?
11. How do you typically use marijuana or cannabis?
  a. Smoking, edibles, vaping etc
12. Do you ever use any marijuana or cannabis products like Delta 8, Delta 10, or THC-O?
  a. What is that like in comparison to typical marijuana/cannabis?
13. Do you use marijuana or cannabis for a specific purpose?
  a. Sleep, pain, mental health
14. How does marijuana or cannabis affect your use of alcohol or any other substance other than marijuana/cannabis?
  a. PROBES: Does using marijuana or cannabis help you avoid other substance use?
  b. PROBES: When you use marijuana or cannabis do you have any cravings for other substances?
15. Do you use marijuana or cannabis with others, like sharing joints or blunts?
  a. **If yes,** what is that usually like? Do you prefer to use it with others?
  b. **If no**, do you prefer to use it alone? Is there a reason why?
16. How would you describe marijuana or cannabis use in relation to your treatment goals?
  a. PROBE: Has using marijuana or cannabis been a positive for your treatment? A negative? Mixed?
17. Where do you usually get your marijuana or cannabis from?
  a. PROBE: Do you have a marijuana or cannabis card? Do you usually get it from people other than a retail store? What is that like?
18. Do you think people in treatment for substance use can use marijuana or cannabis safely?
19. ***Is there anything else you’d like to say about marijuana/cannabis or using marijuana or cannabis during treatment before we end the interview?***
